# Fine-grained temporal coding of visually-similar categories in the ventral visual pathway and prefrontal cortex

**DOI:** 10.3389/fpsyg.2013.00684

**Published:** 2013-10-17

**Authors:** Yang Xu, Christopher D'Lauro, John A. Pyles, Robert E. Kass, Michael J. Tarr

**Affiliations:** ^1^Machine Learning Department, Carnegie Mellon UniversityPittsburgh, PA, USA; ^2^Center for The Neural Basis of Cognition, Carnegie Mellon UniversityPittsburgh, PA, USA; ^3^Department of Behavioral Sciences and Leadership, United States Air Force AcademyCO, USA; ^4^Department of Psychology, Carnegie Mellon UniversityPittsburgh, PA, USA; ^5^Department of Statistics, Carnegie Mellon UniversityPittsburgh, PA, USA

**Keywords:** visual category learning, categorization, ventral visual pathway, prefrontal cortex, cortical time course, decoding, MEG, human neuroscience

## Abstract

Humans are remarkably proficient at categorizing visually-similar objects. To better understand the cortical basis of this categorization process, we used magnetoencephalography (MEG) to record neural activity while participants learned–with feedback–to discriminate two highly-similar, novel visual categories. We hypothesized that although prefrontal regions would mediate early category learning, this role would diminish with increasing category familiarity and that regions within the ventral visual pathway would come to play a more prominent role in encoding category-relevant information as learning progressed. Early in learning we observed some degree of categorical discriminability and predictability in both prefrontal cortex and the ventral visual pathway. Predictability improved significantly above chance in the ventral visual pathway over the course of learning with the left inferior temporal and fusiform gyri showing the greatest improvement in predictability between 150 and 250 ms (*M*200) during category learning. In contrast, there was no comparable increase in discriminability in prefrontal cortex with the only significant post-learning effect being a decrease in predictability in the inferior frontal gyrus between 250 and 350 ms (*M*300). Thus, the ventral visual pathway appears to encode learned visual categories over the long term. At the same time these results add to our understanding of the cortical origins of previously reported signature temporal components associated with perceptual learning.

## 1. Introduction

Objects from visually-similar categories can be difficult to distinguish, but human observers can make accurate category judgments within a fraction of a second, a visual skill that is perfected by learning and experience (Gauthier et al., 2009). Beyond the case of face individuation where each category is mapped to an identity, the more general ability to assign categories to visually-similar objects has important consequences in our natural environment. For example, distinguishing between ripe or poisonous berries, wet or icy roads, or Retrievers or Rottweilers, all necessitate placing one collection of visually-similar objects into a common category, yet keeping that category distinct from another collection of objects that are not only similar to one another, but to the objects in the first category. This sort of categorization is often referred to as “subordinate” to differentiate from “basic-level” categorization in which there are significant visual differences supporting placing objects into one category or another (e.g., pigs vs. airplanes). Moreover, it is often assumed that subordinate-level categorical decisions will incur a larger cost in response time as compared to basic-level categorical decisions–indeed, this functional definition is often used to ascertain whether a given category is considered basic or subordinate (Rosch et al., 1976). At the same time, this response time differential can be minimized through experience in that visual “experts” exhibit an entry-level shift whereby subordinate categorization for domains of interest becomes just as fast as basic-level categorization (Jolicoeur et al., 1984; Tanaka and Taylor, 1991). For example, for bird experts, distinguishing between different species of birds, all nominally members of the same basic-level category, is likely to be just as fast as in telling a bird from a chair. Thus, we can view becoming proficient at categorizing visually-similar objects as an instance of perceptual expertise with subordinate category discriminations. While it is understood that both the ventral occipito-temporal visual cortex, in particular the ventral visual pathway (VVP), and the prefrontal cortex (PFC) are involved in such visual categorization tasks, there is no strong consensus on the relative roles of these neural substrates. Moreover, once specific subordinate-level categorization proficiency has been acquired, there is still a poor understanding of the precise timing of the contributions of the VVP and PFC during the on-line discrimination of visually-similar objects.

To better characterize the roles of the VVP and PFC in the categorization process, we use magnetoencephalography (MEG) to unravel the cortical time course in visual category learning in order to evaluate two prominent, yet competing, theories. The first approach, which we refer to as the “dominant PFC viewpoint,” emphasizes the role of prefrontal cortex (PFC) in categorization and proposes the VVP to be sensitive to visual feature differences but agnostic as to category memberships (Jiang et al., 2007; Seger and Miller, 2010). For example, Jiang and colleagues (2007) found that categorization training induces category-level changes in lateral PFC but only continuous shape-level changes in lateral occipital cortex (LOC). Related work in non-human primates likewise suggests a similar distinction between PFC and inferiortemporal cortical neurons (Miller et al., 2002; Freedman et al., 2003; Meyers et al., 2008). These and other data paint a picture of PFC as the neural substrate supporting category learning and the VVP as the neural substrate providing the undifferentiated (with respect to category) perceptual input that the category-knowledgeable PFC utilizes.

An alternative approach, which we will refer to as the “complementary PFC viewpoint,” suggests that the VVP and PFC play complementary roles in categorization (Mishkin et al., 1983; Goodale et al., 1994; Ungerleider and Haxby, 1994; Bar et al., 2005; Folstein et al., 2012a). Under this view, the VVP exhibits category boundary sensitivity (Sigala and Logothetis, 2002; de Baene et al., 2008) and the PFC provides early top-down categorical inferences that facilitate initial learning of category-relevant feature dimensions (Fenske et al., 2006). Learning and reinforcement progressively instantiate these stimulus dimensions within the VVP; that is, the VVP becomes increasingly sensitive to learned category boundaries as the high-dimensional stimulus space is mapped. This is clearly seen in fMRI for highly overlearned, “expert” domains in which the VVP shows spatially localized, differential responses to subordinate-level categories such faces (Kanwisher et al., 1997), novel objects (Gauthier et al., 1999; Op de Beeck et al., 2006), birds and cars (Gauthier et al., 2000). Similarly, event-related potential (ERP) has consistently revealed category sensitivity in the VVP-sourced *N*170 component (Tanaka and Curran, 2001) and, in several studies of visual expertise, has been localized to posterior occipito-temporal areas (Rossion et al., 2003). Again, as with the fMRI results, this category sensitivity for domains of expertise has been found for both real world (Tanaka and Curran, 2001) and lab-trained experts (Scott et al., 2006).

Some of the discrepancies between results supporting these two approaches may be accounted for by differences in stimulus-morphing procedures used in different experiments. In particular, research supporting a dominant PFC view has typically used a more difficult to learn type of morph space (i.e., blended morphspace). In contrast, research supporting the complementary PFC view has typically relied on a grid morph space (for a thorough consideration of the topic, see Folstein et al., 2012a). This raises the possibility that morphing procedures are actually driving the apparent differences in the role of the PFC for these experiments: the extremely difficult morphspaces require more PFC intervention for participants to map category boundaries, which in turn supports a dominant PFC viewpoint, while the more comprehensible morphspace experiments find that VVP areas are capable of instantiating category boundaries in and of themselves, supporting a complementary PFC viewpoint. As such, perceptually homogenous subordinate categories that have clear decision boundaries, may serve as an ideal test for comparing these views of the PFC's role in categorization. In our experimental paradigm, category membership is never as indeterminate as it would be in the blended morphspace seen in dominant PFC studies, but accurate categorization is still challenging, due to the subtle differences in category features. This design retains the difficulty of blended morphspaces with the predictability of grid morphspaces. Thus, this experiment has the potential to resolve some of the reported differences between the two approaches on the magnitude of the role that VVP plays in the context of subordinate categorization.

To evaluate both approaches, we studied human cortical activity while participants learned to discriminate between two novel and highly-similar visual categories. We hypothesized that although both the VVP and PFC would be involved in the categorization process, their roles would differ during different phases of learning, which is more consistent with a VVP-PFC complementary viewpoint. More specifically, we predicted that the VVP would acquire categorical representation as learning progressed to the point where the category boundaries are better distinguished by participants. In contrast, we predicted that PFC would play a more significant role in category encoding in the initial phases of learning, during the early formation of categorical representations, but that this role would diminish later in learning. With respect to these predictions, the differential roles of the VVP and PFC have been explored by Bar et al. (2005), who found, in a visual recognition task that PFC responses both temporally preceded those in the VVP and were more sensitive to low spatial frequencies. They hypothesized that PFC may be involved in providing early inferences regarding object identities that are subsequently refined by further visual processing within the VVP. Our predictions are related to this hypothesis, but are critically different in two important aspects. First, we focused on categorization instead of individual object recognition—it remains unclear whether the VVP and PFC both play a role in discriminating between visually-similar categories. Second, we investigated the change in response for the VVP and PFC over the course of category learning as opposed to investigating only the end point of learning. In particular, this latter manipulation allowed us to monitor how neural coding of categories change over time, which should offer a better means for better elucidating the functional roles of both PFC and the VVP.

**Figure 1:**
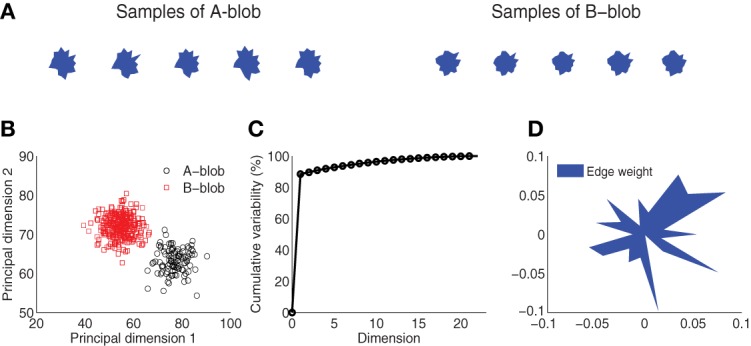
**Visual stimulus design. (A)** Blob samples from *A* and *B* categories. **(B)** Projection of *A* and *B* blobs in two principal dimensions via principal components analysis. **(C)** Cumulative variability accounted for in the principal dimensions. **(D)** Normalized edge weights from the first principal dimension.

To pursue these goals, we created two novel, visually-similar shape categories inspired by the stimuli used by Krigolson and colleagues (2009). Figure 1A illustrates the stimulus image space, showing five samples from each of the two categories. Each of these blob-like exemplars is unique and represents a jittered version derived from one of the two prototypes located at the center of the space of samples forming each category. Although these exemplars are perceptually similar with small differences in the edge contours, a distinct category boundary is embedded in the overall space, as illustrated by two distinct clusters shown in Figure 1B. Participants were trained to discriminate between these two “blob” categories in a feedback-driven categorization task in which we monitored neural activity using MEG. At a fine temporal scale, MEG's millisecond temporal resolution afforded us the ability to investigate how different cortical regions embed discriminative information about the blob categories over time. At a coarser temporal scale, we were able to explore how the encoding of this category information evolves during the course of learning, particularly with respect to categorical representations in the ventral and occipito-temporal visual and prefrontal cortices.

## 2. Materials and methods

### 2.1. Ethics statement

All experimental procedures were approved by the Institutional Review Boards at Carnegie Mellon University and the University of Pittsburgh. All participants gave written informed consent and were compensated financially for their participation.

### 2.2. Participants

Ten right-handed participants (4 females and 6 males) aged between 17 and 35, recruited from the Pittsburgh area, were run in the experiment. Participants were financially compensated for their participation. Two of the participants ran in experimental sessions in which trigger failures meant that the timing of individual trials could not be retrieved, so the data for these two participants was discarded. One participant was unable to correctly learn the blob category boundaries, exhibiting near-chance categorization accuracy throughout the experimental session, so the data for this participant was likewise discarded. Thus, the results reported here are based on the remaining seven participants.

### 2.3. Stimulus design

The visual stimuli were generated from two novel artificial categories, *A* and *B*. Each category was defined around a prototype “blob” that corresponded to the center of a space of blob exemplars (Figure 1A). Within each category, 300 unique blob exemplars were generated from a parameterized distribution. Each blob was the result of a random two-dimensional polygon with 20 edges (or dimensions) similar to the design used by Krigolson et al. (2009). The edges were defined as distances of proportion 30–70% of the distance between an origin and 20 vertices uniformly distributed around a unit-length circle. To control for statistical variability, blobs were generated from a multivariate Gaussian distribution specified for each category, where the mean of the distribution is the 20-dimensional vector of the prototype, and the covariance is a diagonal matrix with variance in each dimension proportional (20%) to the difference in edge distances across the two exemplars. This ensures samples within each category vary slightly from each other but remain distinct from the other stimulus category. Figure 1A shows several samples drawn from each of the two categories. This design yields a distinct category boundary which is illustrated by the two separate blob distributions as projected into a space spanned by the first two principal components of a PCA (Figure 1B). A comparison of the number of dimensions to cumulative variability establishes that the greatest variation (~90%) among the blob samples is captured in one to two dimensions (Figure 1C). Finally, Figure 1D illustrates the normalized weight that each edge shares in the first principal dimension. A lengthier edge accounts for more variability in this dimension and hence it is more likely to be used as discriminative feature for visual categorization.

### 2.4. Experimental procedures

The experiment involved a trial-by-trial feedback-driven visual category learning task where the participants' task was to discriminate between the two blob categories. Each experimental session consisted of 600 trials that included randomized presentations of 300 unique *A*-blobs and 300 unique *B*-blobs. The session was divided into five equal blocks of trials with brief self-paced breaks between each block to reduce fatigue. The sequence of *A* and *B* blobs was permuted for each participant and the number of presentations of stimuli from each category was balanced during each block.

Each trial began with a machine-synthesized random auditory label of “A” or “B” (630 ms) transmitted via non-magnetic ear-plugs while the participant visually fixated on a centered cross. A projector was used to back-project stimuli on a non-magnetic screen (58 × 81 cm) to display all visual stimuli. After an extended 120 ms fixation, either an *A*-blob or *B*-blob exemplar was displayed at the center of the screen (subtending a visual angle of approximately 3.4 degrees both vertically and horizontally) for a brief interval of 750 ms. During the period while the blob was displayed, the participant responded with a finger press to indicate whether the audio category label matched the blob category (“yes” or “no”). For example, if the participant heard the label “A” followed by a visually-presented “B” blob, the participant would press a button to indicate “yes,” match, or “no,” a mismatch. The “yes” and “no” labels were displayed along the left or right bottom corners of the screen with their positions counterbalanced for each experimental session. A glove response pad was used to allow participants to respond with finger presses with minimal wrist movement. Shortly after response, the participant would receive on-screen feedback after a jittered interval of 150–300 ms: “correct,” “wrong,” or “too slow” were displayed in the center of the screen for 750 ms to indicate the correctness of their response. Participants had to respond within the 750 ms window to avoid the “too slow” feedback. The intertrial interval was 500 ms before the next trial began.

Our experimental procedure is similar to the study by Krigolson et al. (2009) with two important distinctions. First, we used an audio label as a prompt for each category to be matched to the subsequent visual presentation of a blob exemplar, whereas in Krigolson et al. (2009) each blob stimulus was simultaneously shown below a randomized written label showing either the letter “A” or “B.” Their trial design made it difficult to determine whether the observed categorical visual responses were driven by the visual letter or the blob stimulus. Second, Krigolson et al. (2009) were equally interested in categorization and error-driven learning, so they continually shortened stimulus presentation to ensure an adequate number of errors for analysis. In contrast, our primary interest was in understanding visual category learning, therefore we maintained a stable visual presentation time throughout our experiment.

### 2.5. MEG data acquisition and preprocessing

Using MEG, we recorded cortical activity while participants were trained to discriminate between the two blob categories. All experiments were conducted in an electromagnetically shielded room with participants seated comfortably and head-fixed throughout the session. Neural data were recorded using a 306-channel whole-head MEG system (Elekta Neuromag, Helsinki, Finland). The system has 102 channels where each is a triplet of a magnetometer and two perpendicular gradiometers.

MEG signals were sampled at 1000 Hz. Four head position indicator coils were placed on the scalp to record relative head positions to the MEG machine at each session. Electrooculography and electrocardiography were recorded by additional electrodes placed above, below and lateral to the eyes and at the left chest, respectively. The coil and electrode signals were used to correct for movement and artifacts throughout the experiments, the MEG signals were bandpass-filtered between 0.1 and 50 Hz to prevent power-line interference at 60 Hz, and signal projection methods were used to remove artifacts such as heart beats. Any delay in the visual display of stimuli on the screen was measured by photodiodes and was corrected for in all reported results. For all of our analyses, we focused on the 400 ms period after visual stimulus onset and prior to the participant's categorization responses. The baseline defined as 120 ms prior to the onset of the blob stimulus was removed for each trial to account for signal drift.

Cortical source estimates were computed using the Minimum Norm Estimates (MNE) (Hamalainen et al., 1993) in MNE Suite software (http://www.nmr.mgh.harvard.edu/martinos/userInfo/data/sofMNE.php). Source dipoles were evenly distributed (5 mm separation between neighboring sources) with orientations fixed normally to the cortical surface. Surface brain models for each individual participant were constructed by Freesurfer software (http://surfer.nmr.mgh.harvard.edu/) from structural magnetic resonance imaging scans acquired at the Scientific Imaging and Brain Research Center at Carnegie Mellon University (Siemens Verio 3T, T1-weighted MPRAGE sequence, 1 × 1 × 1 mm, 176 sagittal slices, TR = 1870 ms, TI = 1100 ms, FA = 8 degrees, GRAPPA = 2). Based on the neural anatomy of each individual participant, 24 ventral visual and prefrontal cortical regions containing multiple source dipoles were identified from Freesurfer segmentation using the Desikan-Killiany Atlas (Desikan et al., 2006).

### 2.6. MEG sensor-space analysis

A multivariate Hotelling's *t*-test was applied across the MEG time series data to evaluate whether MEG sensor signals carry information capable of discriminating between categories *A* and *B*. At each time point, a multi-dimensional vector was defined as the ensemble signal from 102 scalp magnetometers averaged within a 10 ms window (the time-averaging was performed by taking the mean within a moving window of 20 ms in step of 10 ms along the time course). This vector was then collected for each single trial where a blob exemplar was presented. All trials were divided into two groups based on the category membership of the presented blob stimulus in each trial for the *t*-test. To assess whether the multivariate sensor signal is identical under *A* and *B* groups (null hypothesis), the high-dimensional vectors were first mapped into a lower-dimensional space via principal components that preserved at least 99% signal variability prior to the test. This ensures a non-singular inversion in estimating the covariance matrices in the *t*-tests. The resulting projected vectors from all trials were subsequently evaluated with the Hotelling's *t*-test. The computed value was expressed in terms of a χ^2^ statistic at each time point, and it was repeatedly applied through the entire time course between 0 and 400 ms after the visual onset.

### 2.7. MEG source-space analysis

Similar procedures were applied to the MEG source space. Anatomically bounded regions in the ventral visual pathway and prefrontal cortex were first defined by the segmentation result from Freesurfer. Because each region contained multiple dipoles, a multivariate Hotelling's *t*-test was performed over time to evaluate whether dipoles within each cortical region discriminated trials containing *A* or *B* blobs. At each time point, a multidimensional vector was constructed by the ensemble of cortical dipole amplitudes averaged in 10 ms windows. This vector was then reduced via principal components analysis to lower dimensions that capture 99% variability (again to ensure non-singular inversion in the covariance estimation). The resulting projected vectors from all trials were evaluated with the Hotelling's *t*-test at each available time point. The analysis was repeated among first 100 trials and final 100 trials separately to compare the neural discriminability of visual categories at different stages in the learning process.

An excursion test (Xu et al., 2011) was used to evaluate the significance of the discriminative time course in source space. This followed a number of steps. First, discriminative time course was thresholded and only contiguous time points that exceeded the threshold were proposed as potential regions of interest. Contiguity was satisfied if any of the immediate neighbors of a given point in time also passed the threshold—this procedure helped to prune isolated events that are likely to occur due to chance. This same procedure was then applied to the same data multiple times (100-fold permutations), but in each case, category labels were shuffled—this provided a baseline measure, or a null distribution. A *p*-value was then computed using a standard permutation test by comparing the discriminability statistics within the proposed regions of interest to those in the permuted data following procedures described in Xu et al. (2011).

Logistic regression was used to predict blob categories from cortical time course activities at predefined time windows. Within each of 24 anatomically defined cortical regions, time courses of all available cortical dipoles were averaged across time windows 50–150, 150–250, 250–350 and 0–50 (baseline) post-stimulus, respectively. The predictive decoding analysis was then performed within each of these windows. First, ensembles of cortical dipole amplitudes were collected for 100 trials in earliest and final phases of the learning session separately. For each phase, a leave-one-trial-out cross-validation was used to predict the category membership of blob presented at a single held-out trial. Specifically, the multidimensional ensemble of dipole amplitudes for each anatomical region were projected to a low-dimensional space via principal components that captured 99% variability. Then, at each round of cross validation, a logistic regression classifier was used to predict the blob category in an unseen held-out trial given logistic weights estimated from all remaining trials. This procedure was repeated for all trials until every trial was predicted, and the overall accuracy was reported based on the percentage of trials where the classifier correctly predicted the blob category.

## 3. Results

### 3.1. Behavioral category learning performance

**Figure 2:**
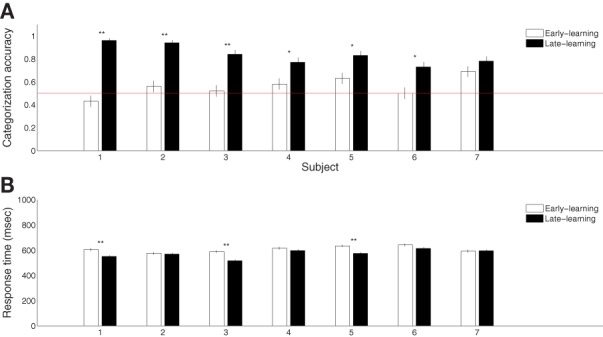
**Summary of behavioral category learning performance. (A)** Categorization accuracies during the early (first 100 trials) and late (final 100 trials) periods of the learning experiment. **(B)** Reaction times during trials from the same periods. “^*^” and “^**^” indicate significance at *p* < 0.05 and *p* < 0.005, respectively, with Bonferroni corrections.

Seven participants successfully learned the blob categorization task. Figure 2A shows the individual categorization accuracies in the first and final 100 trials (error bars indicate standard errors of the means), representing behavioral performance during early and late stages of learning. All but one participant improved significantly (*p* < 0.05 from binomial tests with Bonferroni corrections) over the course of learning. The remaining participant also improved, although the improvement was only marginally reliable (*p* = 0.07). However, all participants were able to categorize the blobs significantly above chance rate 50% (*p* < 0.01 from *t*-tests) with an average terminal accuracy of 83% for the late stage of learning. Figure 2B shows the mean reaction times for the early and late stages of learning (error bars indicate standard errors of the means). Only three subjects showed significant reduction in the reaction time (*p* < 0.005 from *t*-tests with Bonferroni corrections). This was expected because the 750 ms-deadline period was sufficiently short for a combined perceptual and motor response for some participants.

### 3.2. Category-discriminative time course in MEG sensor space

Given that our participants successfully learned the two visual categories, our next step was to assess whether category memberships can be reliably discriminated from MEG sensor data. We expected the recorded sensor data to differentiate trials in which participants recognized blobs from category *A* as compared to category *B*. To evaluate this proposal, we performed Hotelling's *t*-tests with dimension-reduced magnetometer signals and computed category discriminability (χ^2^ statistic) over time using all available trials partitioned into *A* and *B* categories. To obtain a chance-level distribution for comparison, we also applied this procedure to trials with shuffled category labels (100-fold permutations) for each individual subject.

**Figure 3:**
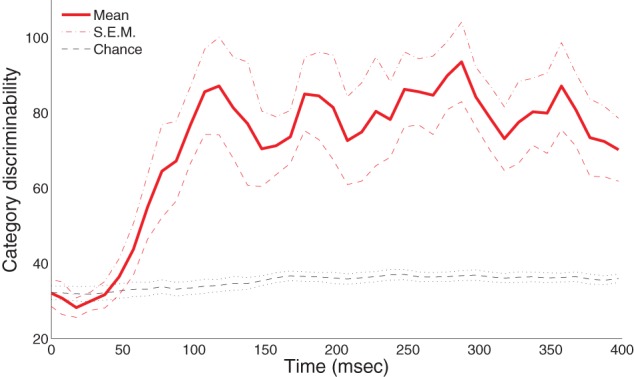
**Category-discriminative time course in MEG magnetometers**. Group-level category-discriminabilitive time course (visual stimulus onset at 0 ms) compared against pooled chance-level time course computed from trials with shuffled category labels.

**Figure A1:**
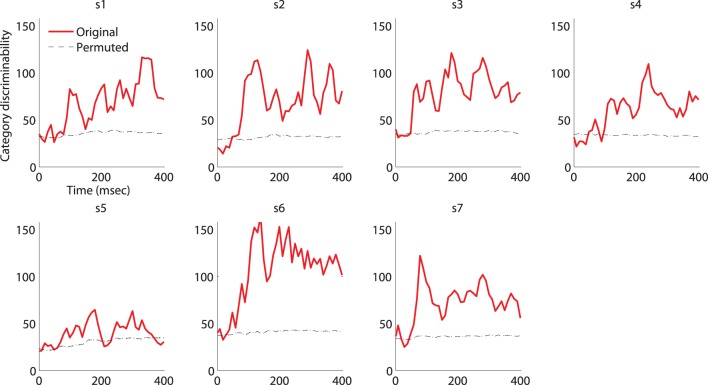
**Category-discriminative time course in MEG magnetometers**. Individual category-discriminative time course (visual stimulus onset at 0ms) compared against discriminative time course computed from trials with shuffled category labels (100 permutations). The 95% confidence intervals of the permuted time course (almost) overlap with the mean.

Figure 3 shows the group-level statistics. We were able to reliably discriminate the *A* and *B* blob categories within the half-second period after visual onset in a single trial. In particular, the mean category discriminability rises post-50 ms and is highly separable from the chance-level after 100 ms. To assess the significance of these results, we applied an excursion procedure similar to (Xu et al., 2011) that compares the temporal statistics from the original data (without permutation) with the permuted statistics. We found that category discriminability is statistically significant post-100 ms for all subjects (combined *p* < 1.8 × 10^−8^ from Fisher's method; *p* < 0.01 from individual-based excursion tests). Figure A1 in the Appendix shows the time course for each individual subject.

### 3.3. Category-discriminative time course in the ventral visual pathway and prefrontal cortex

Our previous analysis demonstrates that the time course in MEG sensors contains significant category information in aggregate, but it does not address the question of localizing which brain regions are the sources of this information or how these sources may change with learning. To evaluate our hypotheses regarding the relative roles of the ventral visual pathway and the prefrontal cortex, we used similar methods to compute category-discriminative time series in MEG *source* space. In particular, we focused on anatomically-defined regions in ventral occipito-temporal visual and prefrontal cortices.

To test whether the ventral visual pathway is capable of learning and discriminating exemplars from visually-similar categories, we compared time courses in related cortical regions during both the early and late stages of learning. Similar to our sensor-space analysis, a category-discrimination time course in source space was computed by performing multivariate Hotelling's *t*-tests from cortical dipole activities across time. To distinguish trials in the early and late stages of learning, tests were performed for the 100 earliest and the 100 latest trials separately with equal numbers of *A* and *B* blobs presented.

**Figure 4:**
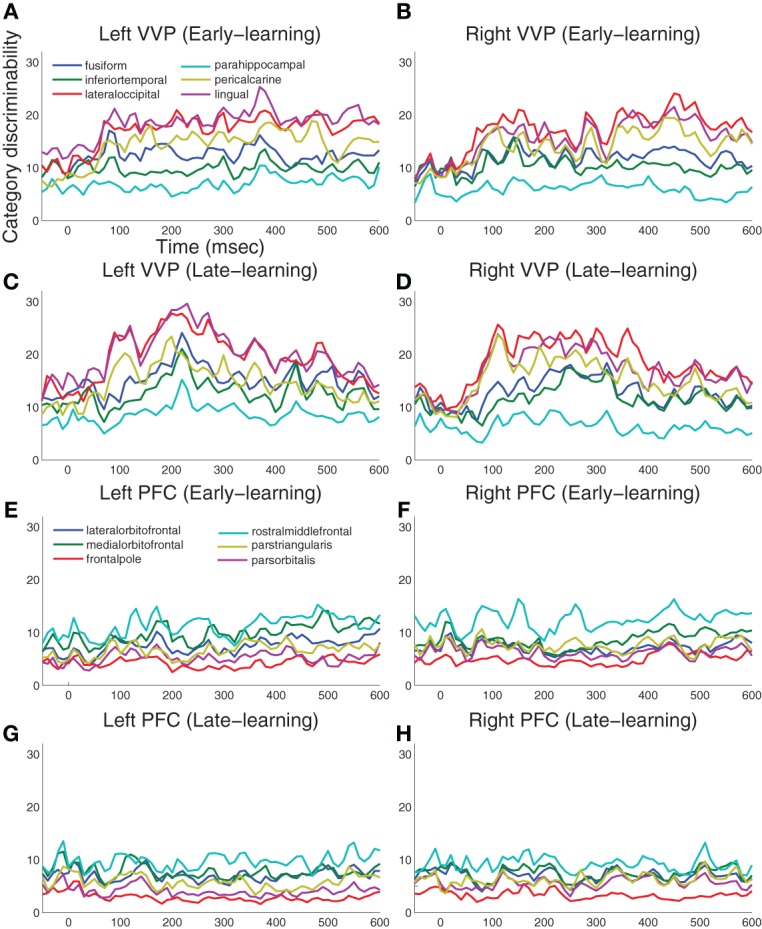
**Category-discriminative time courses in ventral visual and prefrontal cortices. (A)** Group-level discriminative time courses in right-hemispheric VVP contrasting dipole responses in trials containing *A* and *B* blob categories during early learning. **(B)** Discriminative time courses in left-hemispheric VVP regions during early learning. **(C,D)** *P*-value time courses in VVP regions from left and right hemispheres during late learning. **(E–H)** Discriminative time courses in PFC regions under similar conditions.

Figure 4 summarizes the results for 12 visual cortical regions and 12 prefrontal regions in both left and right hemispheres. During early learning as illustrated in Figures 4A,B, we observed that category discriminability rises at approximately 100 ms post-stimulus in both hemispheres. During late learning as illustrated in Figures 4C,D, we observed that category discriminability also rises at approximately 100 ms, but discriminability peaks post-200 ms in the lingual, lateral-occipital and fusiform gyri in the left hemisphere. This time window agrees roughly with *N*250 as previously reported in Krigolson et al. (2009), except here we provided better localization of its sources in the cortex. In comparison, we observed relatively scarce discriminability in prefrontal cortex throughout time course and learning as illustrated in Figures 4E–H.

**Figure 5:**
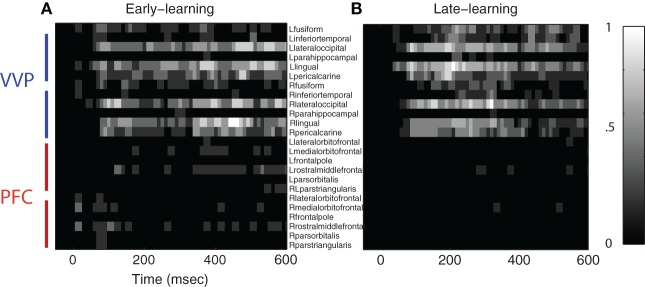
**Regions of interest in ventral visual and prefrontal cortices after excursion tests. (A)** Group-aggregated regions of interest during early learning. **(B)** Group-aggregated regions of interest during late learning. The color bar indicates the tally (normalized across subjects) where a specific cortical region at a time point passes the excursion test.

To assess the significance of the category-discriminative time course, we performed an excursion test following Xu et al. (2011). Specifically, for each subject, we obtained regions of interest by thresholding the time course at 20 and kept contiguous time points that passed the threshold. We evaluated the significance for each subject by comparing the discriminability statistics within the proposed regions of interest against the statistics within regions found from the permutated data (100 folds)—this yielded a global *p*-value. Figure 5 shows the temporal regions of interest pooled across subjects (combined *p* < 1.8 × 10^−8^ from Fisher's method; *p* < 0.01 from individual-based excursion tests). These results show that category information flows primarily in the bi-lateral occipital, lingual, pericalcarine, fusiform and inferior-temporal gyri during both early and late learning, suggesting that the VVP acquires discriminability of novel, visually similar categories during learning.

**Figure A2:**
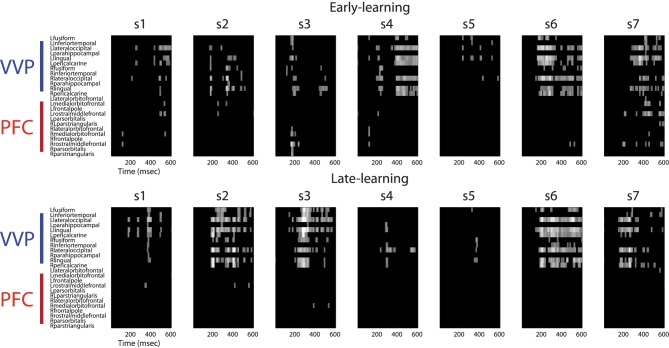
**Regions of interest in ventral visual and prefrontal cortices after excursion tests**. The upper row shows regions of interest during early learning for each individual subject. The bottom row shows regions of interest during late learning. Regions of interest (in bright color) for each subject was validated using an excursion test that yielded a *p* < 0.01 with 100-fold permutations.

Figure 5 also shows that regions of interest in prefrontal cortex are more sparse in comparison with those in the VVP. In particular, whereas temporal coding appears in prefrontal cortex during early learning, it decreases in late learning, suggestive of a diminished role of prefrontal cortex. Figure A2 in the Appendix shows that such a pattern is consistent across all subjects. Our current set of results, however, does not rule out the possibility that coding in PFC becomes more sparse over time (e.g. Meyers et al., 2008) or that it could be generated from a deep source which is difficult to detect with MEG.

### 3.4. Predicting categories from cortical activity

To this point, our analyses have explored category discriminability across a continuous time course. These analyses also help identify time windows that appear to offer availability of category-discriminative cortical information. Thus, one question we can ask is how temporal windows differ from one another with respect to what information they carry regarding visual category learning. A similar question may be asked with respect to spatially localized activity: does the ventral visual pathway carry more information regarding subordinate-level visual categories relative to prefrontal cortices?

To address these questions, this next analysis evaluates to what extent the ventral visual pathway and prefrontal cortex are *predictive* of blob categories at the discrete temporal windows of *M*100 (50–150 ms), *M*200 (150–250 ms) and *M*300 (250–350 ms), as well as, critically, how category predictability within these temporal windows changes over the course of learning. We predict that the ventral visual pathway will play a significant role in category learning and representation. In particular, the VVP is expected to acquire an increasing degree of category predictability (more than PFC) during learning.

To test this prediction, we performed a decoding analysis to assess category predictability in the same 24 anatomically-defined regions in the ventral visual pathway and prefrontal cortex used in our earlier analyses. Within each of these regions, we ran held-out predictions regarding blob categories on a trial-by-trial basis using multidimensional cortical dipole activities averaged within the following time windows: *M*100 (50–150 ms), *M*200 (150–250 ms), and *M*300 (250–350 ms), as well as the baseline of 0–50 ms, post-stimulus. This was implemented using a standard leave-one-out cross validation technique which evaluated to what degree category membership of a blob presented in a single trial not part of the training set can be predicted based on region-bounded dipole responses and blob category labels from the remaining trials in the training set. To compare predictability during initial and end-stage learning, as in the previous analysis, this decoding analysis was conducted separately for the first and final 100 trials.

**Figure 6:**
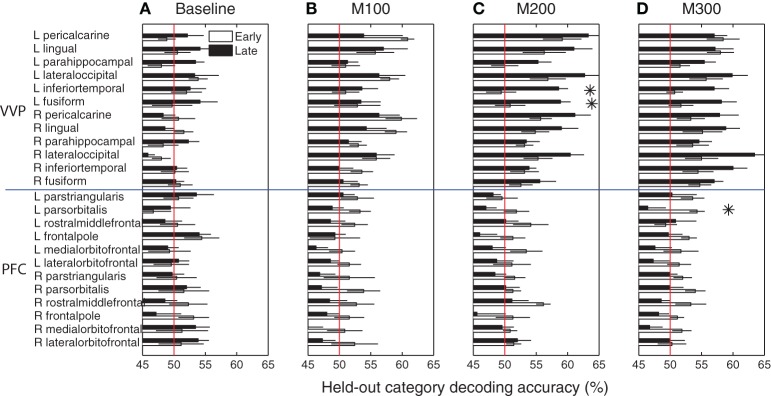
**Category predictive accuracies in ventral visual and prefrontal cortices. (A)** Group-average blob category predictive accuracies in 24 ventral visual and prefrontal cortical regions based on dipole activities in 0–50 ms after onset during early and late learning. **(B)** Decoding accuracies in *M*100 (50–150 ms) window. **(C)** Decoding accuracies in *M*200 (150–250 ms) window. **(D)** Decoding accuracies in *M*300 (250–350 ms) window. Asterisks indicate significant difference (*p* < 0.05) in predictive accuracy between early and late learning.

Figure 6 summarizes blob category-predictive accuracies across all 24 cortical regions and time windows in the early and late stages of learning. At *M*100, the bilateral peri-calcarine gyri, the right lingual gyrus and the left lateral occipital gyrus become highly predictive with respect to blob categories (*p* < 0.005 under *t*-tests), but no significant difference was observed in predictability between early and late learning (*p* > 0.05 under *t*-tests)—suggesting that category predictability in this early time window may not be shaped by category learning *per se*. Within *M*200 and *M*300 windows, across most of visual cortex, predictive accuracies in the late learning stage are considerably better than they were in the initial learning stage.

**Figure 7:**
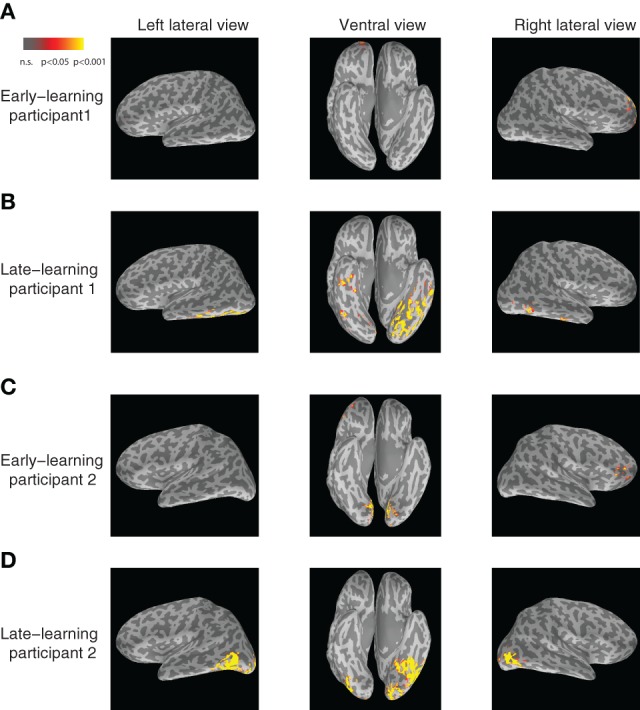
**Visualization of category-discriminative cortical dipoles at *M*200. (A)** Category-discriminative clusters of cortical dipoles from a representative participant during 150–250 ms earlier on in learning. **(B)** Category-discriminative dipoles under similar conditions during late learning. **(C,D)** Category-discriminative dipoles extracted under similar conditions from a second participant.

In particular, the left inferior temporal gyrus (ITG) (*p* < 0.024) and the left fusiform gyrus (FG) (*p* < 0.025) show significant increases in category-predictive accuracy. This pattern suggests that learning plays a greater role in shaping cortical responses at these later temporal stages of processing—confirming our hypothesis that visual cortex encodes and represents subordinate visual categories. To visualize these cortical learning effects, we extracted dipoles that showed reliable differential response (*p* < 0.001) across the *A* and *B* blob categories within the *M*200 window. Figure 7 illustrates the significant discriminability in source dipoles that appeared in the left ITG, the left FG, and the bilateral lateral occipital gyri later in learning. Note that these effects were absent during the initial learning phase of the experiment.

Unlike visual cortex, regions in prefrontal cortex are generally less predictive about blob categories (bottom panels of Figure 6). In addition, these regions are marginally more predictive earlier in learning relative to later in learning, with left pars orbitalis (or inferior frontal gyrus) showing a marginally significant (*p* < 0.05) decrease in predictive accuracy at *M*300. These observations are suggestive that prefrontal cortex plays a greater role in category encoding during learning, but they do not exclude the possibility that learning induces sparse coding in PFC or a more complementary role of PFC that jointly participates category coding with the VVP.

**Figure 8:**
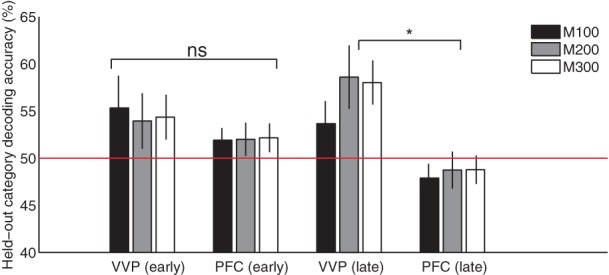
**Cortical category-predictive accuracies**. Pooled held-out category predictive accuracies from ventral visual and prefrontal cortices based on the first and final 100 trials during *M*100 (50–150 ms), *M*200 (150–250 ms) and *M*300 (250–350 ms) after visual stimulus onset. Asterisk indicates significant difference (*p* < 0.005) in predictive accuracy between VVC and PFC at *M*200 and *M*300.

Figure 8 compares the ventral visual pathway and prefrontal cortex at *M*100, *M*200, and *M*300 by pooling predictive accuracies across dipoles within each of these cortical regions. The result suggests that both the VVP and PFC are near chance in predicting the blob categories during initial learning. However, later in learning, the ventral visual pathway becomes significantly more category-predictive than prefrontal cortex at *M*200 and *M*300 (*p* < 0.005 under *t*-tests) but not at *M*100 (*p* > 0.5). Interestingly, we found significant interaction between the VVP and PFC during the three time windows during late learning (*p* < 0.005 under 2 × 3 ANOVA) but not initially during learning (*p* > 0.1). Together, these results support the hypothesis that the VVP and PFC function as complements to one another, suggesting that improved categorization performance over the course of learning is associated with increased predictability post-150 ms for the VVP.

## 4. Discussion

Models addressing the neural basis of visual category learning have focused on the interplay between the ventral visual pathway (VVP) and prefrontal cortex (PFC). However, there has been no clear consensus on the respective roles of these two neural substrates, with some theories taking a dominant PFC view in which category membership is encoded within PFC, while the VVP is sensitive only to visual feature differences (albeit correlated with category membership) (Freedman et al., 2003; Seger and Miller, 2010). In contrast, the complementary PFC view holds that the VVP and PFC play different functional roles at different points in category acquisition—PFC facilitating the learning of category-relevant features during the initial stages of learning, but with the VVP ultimately encoding these featural dimensions so as to become progressively more sensitive to category boundaries (as opposed to purely visual feature differences) (Sigala and Logothetis, 2002; Fenske et al., 2006).

Using MEG which provides superb temporal resolution and good spatial resolution, we conducted a decoding analysis to show that the ventral visual pathway contained the neural information to accurately categorize stimuli with in the first 400 ms after stimulus presentation during a subordinate categorization judgment.

We obtained these findings by using multivariate discriminative and predictive analyses to assess the role of the VVP and PFC during visual category learning. Overall, our data suggested that category-discriminative information is available from the VVP responses in the *M*200 and *M*300 time windows and that responses originating from the left fusiform and inferior temporal gyri acquire a higher degree of discriminability and predictability concomitant with increasing categorization performance. In comparison, we found little evidence that PFC carries significant information about visual categories, but the small sample size encourages a cautious interpretation of this fact.

### 4.1. The functional roles of the ventral visual pathway and prefrontal cortex

As already discussed, our study is in large part based on previous research on visual categorization and category learning using both single and multi-array neural recordings in primates (Freedman et al., 2003; Meyers et al., 2008), and fMRI (Op de Beeck et al., 2006; Folstein et al., 2012b), ERP (Rossion et al., 2002; Wong et al., 2005; Scott et al., 2006; Krigolson et al., 2009), and MEG (Halgren et al., 2000; Liu et al., 2002) in humans. However, to this point, subordinate-level category discrimination at fine-scale temporal resolution with good spatial resolution has primarily been studied at the physiological-level in primates (Freedman et al., 2003; Meyers et al., 2008). Critically, for the majority of these primate-based studies, the stimuli were created in a morphspace where the category boundary could not be clearly specified, an issue that places some constraints on what can be concluded from their results (Folstein et al., 2012a). It is unsurprising that the complicated morphspace studies find more PFC activity than the simpler grid-based design spaces, given the relative difficulty of these two categorization tasks. Meanwhile, Folstein et al. demonstrate that the VVP can instantiate newly-learned category boundary sensitivity when people can focus on diagnostic stimulus dimensions and, essentially, ignore non-diagnostic ones—and that these boundary sensitivites are retained even when task is no longer relevant.

To explore category discrimination in humans, we used a visual stimulus space in which we clustered exemplars to form a distinct category boundary. Moreover, these stimuli were novel to our participants, as such we were able to monitor how the categories became differentiated in the cortex from early to late stages of learning. Our analyses indicated that the measured neural data obtained through MEG tracked the qualitative changes seen in behavioral categorization performance. Our results are consistent with studies that find the VVP to acquire information about stimulus categories, (e.g., Folstein et al., 2012b). More specifically, we found that the lateral occipital complex and the inferiotemporal cortex, possible homologs to the ITC in primates, became significantly more informative with respect to category membership over the course of learning. Contrary to previous findings that support the PFC-dominant theory (Freedman et al., 2003; Jiang et al., 2007), we found that categorical representation is encoded in the human ventral visual pathway even when categories are comprised of perceptually similar items, supporting the idea that visual cortex plays an predominant role in category learning.

Of note, our study is somewhat different methodologically from many other prior category training studies (Op de Beeck et al., 2006; Scott et al., 2006) in that training in our experiment occurred over a single session in which participants are received a training signal in the form of correctness feedback. In contrast, other studies have typically involved a pre-test, a set of training sessions to learn the categories, and a post-test, often including neuroimaging pre- and post- to assess training effects (Gauthier et al., 1999; Op de Beeck et al., 2006). For example, in Op de Beeck et al. (2006), participants completed 10 training sessions in order to learn novel object categories, then performed a color change detection task while fMRI data was collected. Consistent with our present results, they observed a wide range of category-selective responses across the VVP. Interestingly, in this study they observed a change in the spatial distribution of the category-selective responses across training, suggesting that the neural representation of categories changes dynamically with experience. In that our study relied on a single training session, our data cannot address the question as to whether the pattern we observe in VVP would remain stable over further training. Finally, we note that although our single session protocol cannot eliminate the possibility that some of our observed effects are due to attention in that participants necessarily use attentional resources during learning, our results largely converge with these and other studies showing widespread VVP activation with category learning.

Overall, our work suggests that the VVP plays a central role in discriminating visually-similar object categories. However, our results do not rule out the possibility that prefrontal cortex also plays a role in shaping categories—exerting, possibly based on the nature of the categorization task, some top-down influence on visual cortex during learning (Bar et al., 2005). At the same time, our results do not provide evidence for explicit coding of subordinate categories in prefrontal cortex. Beyond our arguments, it is also possible that the coding of categories in PFC is relatively sparse and therefore cannot be detected using the coarse spatial resolution of MEG. Thus, future work is needed to investigate whether sparse codes exist in prefrontal cortex and to address how prefrontal cortex coordinates with visual cortex in representing visual categories during different phases of learning.

### 4.2. The time course of cortical processing during visual category learning

The ERP and MEG literatures contain many proposals about signature waveforms that relate to visual categorization and recognition, the most common ones being time windows at *M*100 (Liu et al., 2002), *N*170 (Rossion et al., 2002) or *M*170 (Liu et al., 2002), and *N*250 (Krigolson et al., 2009)—negative deflecting MEG or ERP components that peak around 100 ms, 170 ms, and 250 ms post-stimulus. Unresolved is how these waveform components relate to coding of visual categories and to what extent they are shaped by learning. To the extent there is any consensus, within the literature the *N*170 has been found to exhibit a greater negative amplitude with increased perceptual experience with a particular stimulus category (e.g., wading birds). Similarly, the *N*250 component has been found to increase with increasing proficiency at identifying individual exemplars within a category. For example, work by Krigolson et al. (2009) found increased negativity at *N*250 after participants learned to discriminate blob stimuli similar to those used here. However, these and related studies focused on negativity as measured by sensor-averaged signals and did not show whether components such as *N*170 and *N*250 actually carry sufficient information to discriminate or predict the learned visual categories.

Along with recent studies that explored visual object decoding using MEG and EEG (Philiastides and Sajda, 2006; Carlson et al., 2011, 2013; Chan et al., 2011), in this study we also went beyond finding raw amplitude differences between categories and asked whether neural signals support category discrimination. In particular, we demonstrate post-stimulus MEG data can both discriminate and predict subordinate visual categories. Moreover, we were able to identify critical time windows by comparing their respective roles in category learning, finding that the *M*100 component is minimally sensitive to learning and seems to be driven largely by low-order visual processes, while the *M*200 and *M*300 components both become more predictive of visual categories by the end of learning. These results are largely consistent with Krigolson et al. (2009) results and support their claim that the *N*250 is a crucial component in characterizing perceptual learning. We further suggest that the *N*250 component is particularly prominent in visual processing and increased category predictability in the ventral visual pathway, possibly due to an interaction between inferiortemporal and fusiform cortices. More generally, these findings are consistent with previous proposals that place the source of *N*170 in posterior inferior temporal cortex (Tanaka and Curran, 2001; Rossion et al., 2002; Wong et al., 2005; Scott et al., 2008) and *N*250 in fusiform areas (Scott et al., 2006)—a claim that might be further resolved by simultaneous MEG and EEG recordings to establish a better correspondence between the ERP and MEG time components.

In interpreting these results, we would like to note that although we posit specific temporal windows at *M*100, *M*200, and *M*300 as playing important roles in category learning, these markers should not be taken as a strict classification or as markers of mechanisms arising from isolated cortical areas. On the contrary, these components are more likely to arise from functional networks driven by a combination of bottom-up and top-down interactions among cortical and subcortical structures (Ashby et al., 1998; Kveraga et al., 2007), where the measured waveforms are manifestations of cortical systems that exhibit the most robust responses. Future work should examine how visual category learning is communicated interactively among cortical and subcortical areas to achieve efficient categorization, as well as how such communication emerges in category learning.

Finally, we should note that although our study focused on cortical dynamics—the domain in which feedforward visual category coding most plausibly occurs—a separate, yet important, aspect of visual categorization involves feedback learning, often propagated through deeper structures such as the basal ganglia and anterior cingulate cortex. While extensive research (Gehring et al., 1993; Ashby et al., 1998; Holroyd and Coles, 2002; Seymour et al., 2004; Holroyd et al., 2005) indicates that basal ganglia and anterior cingulate cortex are crucial in trial-and-error learning and decision making processes such as that employed in our category learning task, detecting neural signals from deep cortical and subcortical structures is typically not feasible using MEG (Hamalainen et al., 1993). For this reason, some category information may also be contained in these neural substrates, but would not be revealed by our analyses due to the depth of these structures and the limitations of the MEG signal.

## 5. Conclusions

In sum, our findings support a complementary PFC view of visual category learning. This view is supported by previous work showing both early PFC influences in object recognition processes (Bar et al., 2005) and category boundary sensitivity within VVP areas (Folstein et al., 2012b). Critically, not only does the VVP carries category-predictive information, but it does so in a time frame that agrees with the predictions of the complementary PFC viewpoint: the VVP increases in its category predictiveness as learning increases. More generally, our work offers an account that uniquely considers *combined* spatiotemporal properties associated with the encoding of subordinate categories, and further, how these properties change over learning. As such, we consider this study to be a starting point for a better understanding of the complex and interactive neural mechanisms underlying visual category learning.

### Conflict of interest statement

The authors declare that the research was conducted in the absence of any commercial or financial relationships that could be construed as a potential conflict of interest.
